# Substantia nigra micro-haemorrhage causing ipsilateral unilateral Parkinsonism and abnormal dopamine transporter scan uptake

**DOI:** 10.1259/bjrcr.20200118

**Published:** 2020-10-08

**Authors:** Aliaa Ghoneim, Christopher Pollard, Alok Tyagi, Ravi Jampana

**Affiliations:** 1 Consultant Neuroradiology, King Abdulaziz University, Jeddah, Saudi Arabia; 2 Department of Neuroradiology, Consultant Neuroradiology, Institute of Neurological Sciences, Glasgow, UK; 3 Department of Neurology, Consultant Neurology, Institute of Neurological Sciences, Glasgow, UK; 4 Department of Neuroradiology, Consultant Neuroradiology, Institute of Neurological Sciences, Glasgow, UK

## Abstract

Parkinsonism is a commonly seen movement disorder syndrome with neurodegenerative and non-neurodegenerative causes. Presynaptic dopamine transporter (DaT) single‐photon emission computed tomography (SPECT) imaging is the most commonly used imaging technique in clinical practice to differentiate degenerative Parkinson’s disease (PD) and PD plus syndromes from other causes such as essential tremor and drug-induced parkinsonism. This can help identify the patients who would benefit from medical therapy due to underlying pre-synaptic dopaminergic deficits. We report a case of unilateral parkinsonism caused by ipsilateral substantia nigra micro-haemorrhage resulting in disruption of the nigrostriatal pathway. This is an unusual case of a 55-year-old male patient who presented with unilateral Parkinsonism a decade after significant head trauma where MRI plays a critical and complementary role in diagnosing complete interruption of the nigrostriatal pathway due to cerebral micro-haemorrhage. The case also beautifully demonstrates the anatomy of the nigrostriatal pathway where a small lesion in the substantia nigra caused complete loss of radioligand uptake in the ipsilateral corpus striatum. Physicians should be aware of the importance of structural imaging in atypical movement disorder cases and, in particular, the routine use of susceptibility-weighted sequences (SWI).

## Introduction

Parkinsonism is a movement disorder defined by the presence of bradykinesia in addition to rest tremor or rigidity.^1^ There are several causes for this movement disorder including neurodegeneration (Parkinson’s disease, multiple system atrophy, progressive supranuclear palsy, and corticobasal degeneration) and non-neurodegenerative conditions (vascular parkinsonism, drug‐induced parkinsonism, and essential tremor).^2^ Diagnosis is usually established based on clinical criteria; however, imaging is sometimes essential to aid the diagnosis. MRI of the brain is a helpful first-line imaging tool to differentiate neurodegenerative parkinsonian disorders from parkinsonism caused by lesions involving the nigrostriatal pathway such as infarcts, mass lesions, and demyelination.^3^ Presynaptic dopamine transporter (DaT) single‐photon emission CT (SPECT) uses a radioactive pharmaceutical which binds to the presynaptic membranes of the terminals of dopaminergic projections. DaT is contemplated as an indicator of dopamine terminal integrity from the substantia nigra in the midbrain to the striatum in the basal ganglia (caudate nucleus and putamen).^4^ An abnormal DaT scan can help differentiate conditions associated with pre-synaptic dopaminergic dysfunction, such as neurodegenerative parkinsonism and vascular Parkinson’s, from essential tremor and drug-induced Parkinson’s.

There are many case reports in the literature describing focal lesions within the substantia nigra affecting the nigrostriatal pathway DaT uptake and causing contralateral hemi-parkinsonism. The causes ranging from lacunar infarcts to Japanese encephalitis^5–7^ have been described. To our knowledge, this is the first case of hemi-parkinsonism caused by ipsilateral substantia nigra micro-haemorrhage resulting in disruption of the nigrostriatal pathway. This emphasises the importance of susceptibility-weighted sequences in imaging patients with parkinsonism especially in the presence of unilateral abnormal DaT scan and normal head CT or structural MRI scans.

## Case presentation

**Figure 1.**
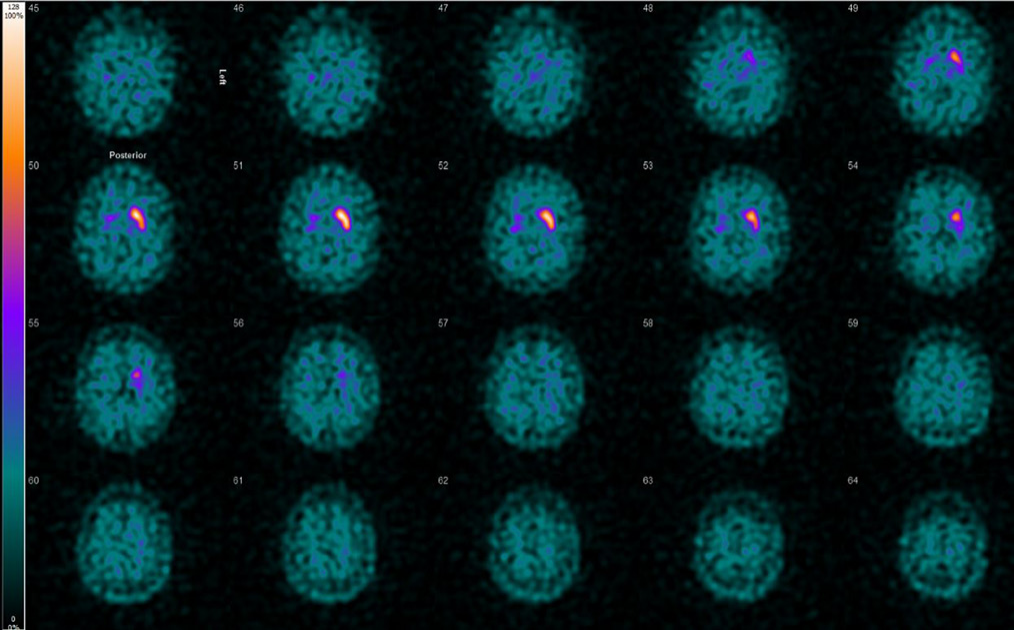
DaT scan showing absent radioligand uptake within the right caudate and putamen.

**Figure 2.**
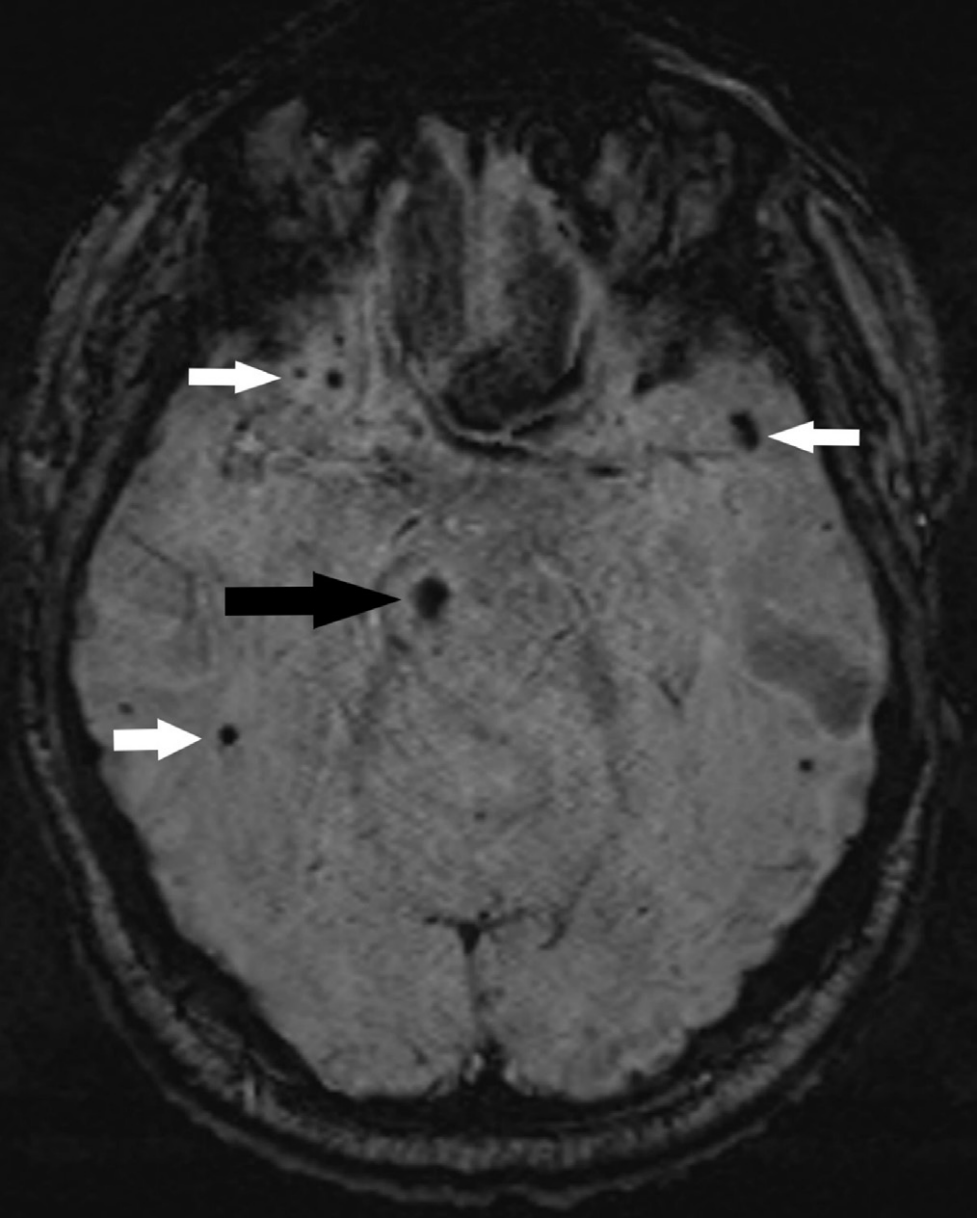
SWI of MRI brain showing haemorrhage within the right substantia nigra (black arrow). In addition to multiple micro-haemorrhages within bilateral frontal and temporal lobes (white arrows).

We present a 55-year-old male patient who sustained a head injury in a road traffic accident about 13 years ago. CT scan of his head at that time showed multiple brain parenchymal haemorrhagic contusions involving the corpus callosum, bilateral frontal, and temporal lobes. He suffered right-sided hemiparesis since then. Seven years later he started to develop gradual worsening of the unilateral hemiparesis along with new onset of rigidity and hyperreflexia, symptoms which are indicative of extrapyramidal syndrome and had worsened along the course of 2 years. He was thus investigated for the possibility of idiopathic Parkinson’s disease. MRI of his head was done and showed encephalomalacia changes involving bilateral superior frontal gyri. The lesion within the left frontal lobe involves the supplementary motor cortex (area 6) which explains the post-traumatic right-sided hemiparesis that he had suffered from for years. No abnormality was seen within the basal ganglia or midbrain to explain the extrapyramidal symptoms. No susceptibility-weighted imaging was obtained at that time. The DaT scan showed complete absence of the radioligand uptake within the right striatum with normal uptake on the left (Figure 1). Whilst asymmetric onset is a clinical hallmark of idiopathic Parkinson’s disease, the marked asymmetry where one side is normal is not a feature of idiopathic Parkinson’s disease and suggests a structural cause. Furthermore, a repeat DaT scan of our patient two years later showed no change in the unilateral right striatum absent uptake. He then went on to have further imaging of his brain this time with the addition of SWI sequence, which showed evidence of old haemorrhage within the right substantia nigra (Figure 2). In addition to multiple micro-haemorrhages involving the corpus callosum, bilateral frontal, and temporal lobes in a pattern in keeping with traumatic brain injury. The haemorrhage within the right substantia nigra explains the absent uptake on the DaT scan in keeping with traumatic damage and resultant disruption of the nigrostriatal pathway.

## Discussion and conclusions

Our patient presented with symptoms of unilateral parkinsonism with ipsilateral absent radioligand uptake on DaT scan within the right striatum. The cause of which was found to be right substantia nigra haemorrhage from a previous head injury. Susceptibility-weighted imaging has long been used in imaging of Parkinson’s disease but was always used to detect iron accumulation in the substantia nigra. An area of particular importance is the dorsolateral substantia nigra called Nigrosome one which typically shows hyperintense signal on SWI, the so-called swallow-tail sign. Several studies have proved that the loss of the swallow-tail sign was both sensitive and specific for Parkinson’s disease.^8^ However, few studies have discussed the SWI importance in evaluating unilateral parkinsonism apart from that.Structural imaging is performed in the presence of abnormal DaT scan uptake to rule out focal abnormalities involving the nigrostriatal pathway. This is of particular importance in patients not presenting with the typical clinical picture of degenerative PD. SWI should always be included in the MRI sequencesto evaluate for the presence of iron accumulation and focal haemorrhages.

The connection between the presence of a substantia nigra lesion and unilateral parkinsonism is well recognised.

The substantia nigra is connected to the ipsilateral motor cortex via connections with the caudate and putamen in the process of movement regulation.^9^ Thus, any lesion along the pathway will cause symptoms on the contralateral side-of the body.

DaT scan can evaluate the integrity of the nigrostriatal pathway from the substantia nigra in the midbrain to the caudate and putamen in the basal ganglia. Nonetheless, most of the information in the literature discusses the contra laterality of the clinical symptoms in relation to the substantia nigra lesion. However, one study in evaluating patients with early-stage unilateral Parkinson’s disease found that changes in the substantia nigra correlated with clinical symptoms on the ipsilateral side-of the body.^10^ Furthermore, the radioligand uptake on DaT scan was significantly reduced in the putamen on the symptomatic side. Whereas the putamen uptake contralateral to the symptomatic side was only slightly reduced or normal. This was attributed to being due to hand dominance. This is in concordance with other studies which support the hypothesis that motor symptoms in unilateral parkinsonism are usually on the hand dominant side.^11^Additionally, a case report published in 1978 described a case with right-sided parkinsonism due to a right substantia nigra infarct confirmed at autopsy.^12^ Further studies are needed to justify the occurrence of clinical symptoms ipsilateral to the substantia nigra abnormalities.

To our knowledge, this is the first case of substantia nigra haemorrhage causing unilateral absent uptake in the right striatum. Moreover, the ipsilateral symptomatology of our patient adds to the uniqueness of our case.

## Learning points

1. Substantia nigra micro-haemorrhage is a rare cause for unilateral parkinsonism.
2. The importance of utilizing structural imaging along with nuclear scans in diagnosing movement disorders.
3. The use of susceptibility-weighted imaging in unilateral parkinsonism.
